# XVI^th ^QTLMAS: simulated dataset and comparative analysis of submitted results for QTL mapping and genomic evaluation

**DOI:** 10.1186/1753-6561-8-S5-S1

**Published:** 2014-10-07

**Authors:** M Graziano Usai, Giustino Gaspa, Nicolò PP Macciotta, Antonello Carta, Sara Casu

**Affiliations:** 1 Settore Genetica e Biotecnologie, Dipartimento per la Ricerca nelle Produzioni Animali, AGRIS-Sardegna, 07040 Olmedo, Italy; 2 Dipartimento di AGRARIA, Sezione Scienze Zootecniche, Università di Sassari, 07100 Sassari, Italy

## Abstract

**Background:**

A common dataset was simulated and made available to participants of the XVI^th ^QTL-MAS workshop. Tasks for the participants were to detect QTLs affecting three traits, to assess their possible pleiotropic effects, and to evaluate the breeding values in a candidate population without phenotypes using genomic information.

**Methods:**

Four generations consisting of 20 males and 1000 females were generated by mating each male with 50 females. The genome consisted of 5 chromosomes, each of 100 Mb size and carrying 2,000 equally distributed SNPs. Three traits were simulated in order to mimic milk yield, fat yield and fat content. Genetic (co)variances were generated from 50 QTLs with pleiotropic effects. Phenotypes for all traits were expressed only in females, and were provided for the first 3 generations. Fourteen methods for detecting single-trait QTL and 3 methods for investigating their pleiotropic nature were proposed. QTL mapping results were compared according to the following criteria: number of true QTL detected; number of false positives; and the proportion of the true genetic variance explained by submitted positions. Eleven methods for estimating direct genomic values of the candidate population were proposed. Accuracies and bias of predictions were assessed by comparing estimated direct genomic values with true breeding values.

**Results:**

The number of true detections ranged from 0 to 8 across methods and traits, false positives from 0 to 15, and the proportion of genetic variance captured from 0 to 0.82, respectively. The accuracy and bias of genomic predictions varied from 0.74 to 0.85 and from 0.86 to 1.34 across traits and methods, respectively.

**Conclusions:**

The best results in terms of detection power were obtained by ridge regression that, however, led to a large number of false positives. Good results both in terms of true detections and false positives were obtained by the approaches that fit polygenic effects in the model. The investigation of the pleiotropic nature of the QTL permitted the identification of few additional markers compared to the single-trait analyses. Bayesian and grouped regularized regression methods performed similarly for genomic prediction while GBLUP produced the poorest results.

## Background

The availability of high-density SNP chips for livestock species allowed the implementation of genome-wide association (GWA) and genomic selection (GS) studies. Many of these studies dealt with quantitative traits of economic interest. For dairy species, milk, fat, and protein yields and fat and protein percentages are of great importance. These traits exhibit genetic correlations. It is reasonable to hypothesize that such a correlation is due to quantitative trait loci (QTL) with pleiotropic effects on more than one trait. On the other hand, it should be remembered that fat content, for example, derives from the ratio between fat and milk quantity. Thus, the pleiotropy between contents and yields could be a mathematical artefact. The assessment of the real pleiotropic nature of a QTL could be extremely important when genomic information is included in marker assisted breeding programs.

Similarly to previous QTL-MAS workshops, a common data set was simulated and made available to participants. It consisted of genomic and phenotypic information for a population organized in medium-sized half-sib families over four non-overlapping generations. The phenotypes were three correlated quantitative traits that mimicked milk and fat yields, and fat content.

Tasks for the participants were: i) to detect QTLs affecting the traits and to assess their possible pleiotropic effects; and ii) to evaluate the breeding values in a candidate population without phenotypes using genomic information.

Fourteen methods for QTL detection and 11 methods for predicting genomic breeding values were proposed by 7 research groups attending the meeting. In this paper, results submitted by the participants will be compared.

## Methods

### Simulation

#### Pedigree

A base population (G0) of 1,020 unrelated individuals (20 males and 1,000 females) was generated. Each of the next four generations (G1-G4) consisted of 20 males and 1,000 females and was generated from the previous one by randomly mating each male with 50 females. Females produced female offspring, except for 20 dams of males, which generated 2 offspring (1 male and 1 female). Generations did not overlap.

#### Genome

The genome consisted of 5 chromosomes, each of 100 Mb size and carrying 4,000 equally distributed SNPs. The 2,040 G0 gametes were constructed from the beginning to the end of each chromosome as follows. At each new SNP *j*, prior allele frequencies were simulated as *f(1)'~N(0.5,0.1) *and *f(2)'=1-f(1)'*. The degrees of linkage disequilibrium (LD) between SNP *j *and the SNPs already assigned, as measured by the *r *statistic [1] and listed in ***r' ***, were sampled as ***r'**~N(r°,0.1) *and their sign were sampled to be positive or negative with probability 0.5. The expected absolute *r *values listed in ***r° ***decayed from 1 to 0 according to the distance between SNPs. The allele carried by gamete *i *at the SNP *j *(*h_ij_*) was sampled with probability *P(h_ij _= x_k_|**r'**,h_i_); *where *x_1(2) _*stands for allele 1(2) and *h_i _*lists the alleles already assigned in *i *. Since the level of LD for distances greater than 1 Mb was assumed negligible, only 40 upstream SNPs were considered. This probability was calculated as:

$$P\left(h_{ij}=x_{k}|r^{\prime },h_{i}\right)=\frac{P\left(h_{ij}=x_{k}\right)P\left(r^{\prime }|h_{ij}=x_{k}\right)P\left(h_{i}|r^{\prime },h_{ij}=x_{k}\right)}{P\left(h_{i}|r^{\prime }\right)P\left(r^{\prime }\right)}$$

where:

$$P\left(h_{ij}=x_{k}\right)=f{\left(x_{k}\right)}^{\prime };$$

$P\left(r^{\prime }|h_{ij}=x_{k}\right)=P\left(r^{\prime }\right)$*, s*ince the effect of a single haplotype on the LD of the population was assumed negligible;

$P\left(h_{i}|r^{\prime },h_{ij}=x_{k}\right)$ depends on the expected frequencies of the haplotypes denoted by *j *and the *l^th ^*loci upstream. The latter was derived from the *r *statistic formula [1]:

$$P\left(h_{i}|r^{\prime },h_{ij}=x_{k}\right)=\overset{40}{\underset{l=1}{\prod }}f{\left(x_{k}\right)}^{\prime }f\left(h_{i\left(j-l\right)}\right)+\gamma {r^{\prime }}_{l}\sqrt{f{\left(x_{k}\right)}^{\prime }\left[1-f{\left(x_{k}\right)}^{\prime }\right]f\left(h_{i\left(j-l\right)}\right)\left[1-f\left(h_{i\left(j-l\right)}\right)\right]}$$

where f(h_i(j-l)_) is the realized frequency of the allele carried by i at j-l locus, $r_{l}^{\prime }$ is the LD between j and j-l, *γ *= 1 if x_k _= h_i(j-l) _and γ=-1 if x_k_≠h_i(j-l)_

$P\left(h_{i}|r^{\prime }\right)=\sum_{k=1}^{2}P\left(h_{i}|r^{\prime },h_{ij}=x_{k}\right)$.

The 2,040 gametes were then randomly combined in 1,020 genotypes (20 as sires and 1000 as dams). For the following generations, meioses were simulated by sampling recombinations according to Haldane's mapping function. Finally, all of the even SNPs were hidden so that the final map consisted of 10,000 SNPs located every 0.05 Mb. All genotypes were provided to the workshop participants, except those of G0 females. The software used to simulated genome data was written in Fortran 90 by the authors.

#### QTL simulation

Fifty QTL positions were sampled from among the even SNPs. The allele substitution effects of the QTLs were drawn from a gamma distribution with scale parameter 5.4 and shape parameter 0.42 [2]. The effects were standardized (*α_sdu_*) and their sign were sampled to be positive or negative with probability 0.5. Three correlated quantitative traits were generated in order to mimic milk yield (T1), fat yield (T2), and fat content (T3=T2/T1). The QTL effects in trait units (*α_T._*) were defined as:

$$\alpha_{T1}=\alpha_{sdu}*\sigma_{T1}$$

$$\alpha_{T2}=\omega \frac{\alpha_{T1}*\mu_{T2}}{\mu_{T1}}$$

$$\alpha_{T3}=\frac{\mu_{T2}+\alpha_{T2}}{\mu_{T1}+\alpha_{T1}}-\mu_{T3}$$

where *μ_T1_, μ_T2 _*and *μ_T3 _*(*μ_T3_=μ_T2_*/*μ_T1_*) were the trait means, and were chosen to reflect those estimated in the Sarda breed of dairy sheep (200 kg, 12 kg, and 0.06, respectively); *σ_T1 _*is the standard deviation of T1 (100 kg); and *ω *is a number which defines the relationship between T1 and T2 at the QTL level. Specifically, *ω *= 1 means that the effects for T1 and T2 deviate equally in mean units generating zero effect on T3. Any other value of *ω *generates a non-zero effect on T3. Thus, the values of *ω *affected the variance of T2 and T3, and the covariance between all the three traits explained by each QTL. In fact, positive and negative *ω *values produce positive and negative covariances between T1 and T2, respectively; if *ω*>1, T3 covaries positively with both T1 and T2; if 0<*ω*<1, T3 covaries positively with T1 and negatively with T2; and if *ω*<0, T3 covaries negatively with T1 and positively with T2. Thus, the set of *ω *values assigned to all the QTLs determines the additive genetic variances and covariances between traits. Values were iteratively assigned to *ω *to obtain the desired genetic variances and covariances.

#### Phenotypes

True breeding values (TBV) for the three simulated traits were calculated as the sum of the additive effects of the 50 QTLs in each individual. Random residuals were drawn from normal distributions with mean zero and trait-specific residual variances to simulate heritabilities of 0.35, 0.35, and 0.50 for T1, T2, and T3, respectively. Correlations between residuals were set to be equal to genetic correlations. The phenotypes available to the participants were individual yield deviations (YD) derived as the sum of the TBVs and random residuals for each trait. All traits were expressed only by females, and were made available for G1 through G3.

### Comparison of methods used by participants

#### QTL detectability

To determine which QTLs were potentially detectable for each trait, we fitted a multiple regression with all 50 QTL genotypes on G1 to G3 females. We assumed that QTLs which could not be identified under the correct model would not be correctly detectable by the participants. A multiple regression model was used in order to exclude spurious effects due to linkage with other large QTLs that might be found in single-locus analyses. A QTL was assumed to be detectable if the p-value for an F-test of the estimated effect was <5 × 10^-6^, corresponding to the Bonferroni correction for an overall significance of 0.05 with 10,000 tests.

#### Methods for QTL detection used by participants

Various methods for detecting genome regions affecting single trait were proposed. Karacaoren [3] suggested a GWA by ridge regression on actual YD (RR_YD) or YD adjusted by a principal components correction for LD structure (RR_YDadj). Riggio and Pong-Wong [4] performed a regional heritability mapping (RHM) approach by fitting a mixed model where the effect of a genomic region (20 SNPs) and the overall genetic background were added as random. Minozzi et al. [5] used a selection analysis implemented in the *randomForest *package and a GWA based on Mixed Model and Regression - Genomic Control (GRAMMAR-GC). Both methods were applied to single (RF_ST; GRM_ST) and multiple trait (RF_MT; GRM_MT) estimated breeding values, and on yield deviations (RF_YD; GRM_YD). Moioli et al. [6] applied a selective genotyping (SG) technique based on the comparison of the allele frequencies of sliding windows of 5 consecutive SNPs in two groups divergent for production. Grosse-Brinkhaus et al. [7] performed the GWA with the Genome wide rapid association using Mixed Model and Regression (GRAMMAR). Demeure et al. [8] applied a linkage analysis (LA) based on a within-half-sibs family linear regression by using QTLmap software. García-Gámez et al. [9] performed a GWA based on a mixed model including the pedigree information as a random polygenic effect and each SNP genotype as a fixed effect with the DMU software package. Moreover, they performed a LDLA analysis where, in addition to the polygenic effect, the QTL was fitted as a random effect with a (co)variance structure that was a function of identity-by-descent (IBD) probabilities between haplotypes.

Three authors investigated the pleiotropic nature of the QTLs using different approaches. Karacaoen [3] performed RR on the two first principal components (PC) extracted from YD and YD adjusted for LD structure. Grosse-Brinkhaus et al. [7] proposed two different approaches. First they performed the GRAMMAR procedure on two principal components extracted from the three traits and, as an alternative, they analyzed the data set with a Bayesian multivariate method implemented in the *snptest *package. Finally, Riggio and Pong-Wong [4] estimated correlations between regional EBVs to evaluate possible pleiotropic effects among traits, when a QTL was found significant for more than one trait.

#### Criteria used for comparing the proposed QTL detection methods

Results of the different studies were compared according to 3 criteria: i) the number of true QTLs detected, ii) the number of false positive QTLs, iii) and the proportion of the true genetic variance explained by the true QTLs detected. In particular, a true QTL was considered mapped when one or more of the submitted positions were within 1 Mb of the actual position of the QTL. All of the submitted positions that did not fit this condition were considered false detections. The proportion of variance explained by the detected QTLs was determined on the basis of the true QTL effects.

#### Methods for genomic prediction

For the analysis of data of the XVI QTL-MAS workshop 11 methods were proposed by two participants (Table 1). All the participants fitted univariate models and did not consider genetic correlations among traits. Pong-Wong [10], proposed a new Bayesian method based on a Horseshoe distribution as a prior for the SNP effect to estimate DGV. They compared this approach with five of the most common methods (Table 1) for DGV estimation that basically differ in the prior distribution of the SNP effects, and submitted the estimates of DGV for T1, T2, and T3 for each proposed method. Ogutu et al. [11] proposed five regularizing (or penalizing) regression methods (RRM) which mainly differ in the penalty function implemented. They also focused on the possibility of grouping predictors using regularization methods with grouped penalties (GRRM) specifically designed to enable group selection in order to account for the potential structure among markers (for example, that arising from the presence of haplotype blocks). Groups of markers were formed by assigning consecutive SNPs systematically to groups of 1, 10, 20,..., 100 SNPs for each of the five chromosomes. The DGV or the parameters used for comparing the methods (see next section) were submitted only for the best-performing grouping strategy within GRRM methods.

**Table 1:** Methods used to estimate Direct Genomic Values (DGV) from genotypic and phenotypic data of the XVI QTL-MAS Workshop

First Author	Methods^1^	Label
Pong Wong	Bayesian Horseshoe	Horseshoe
	Bayes A	BayesA
	Bayes B	BayesB
	Bayes C	BayesC
	Bayesian Lasso	BayesLasso
	GBLUP	GBLUP
Ogutu*	Group Bridge Regression	GBRIDGE
	Group Min Max Concavity Penalty	GMCP
	Group Least Angle Shrinkage & selection operator	GLASSO
	Group smoothly clipped absolute deviation	GSCAD
	sparse group LASSO	sgLASSO

* Ogutu evaluated 5 methods with 10 different group size of predictor each

#### Criteria used for comparing the methods proposed for genomic prediction

Two criteria were used to evaluate the proposed methods: *i*) accuracy of predictions, assessed by Pearson correlation between TBV and DGV (r_DGV_); *ii*) bias of prediction, measured by the slope of linear regression of TBV on DGV (b_DGV_).

## Results

### Simulation

**Figure 1:**
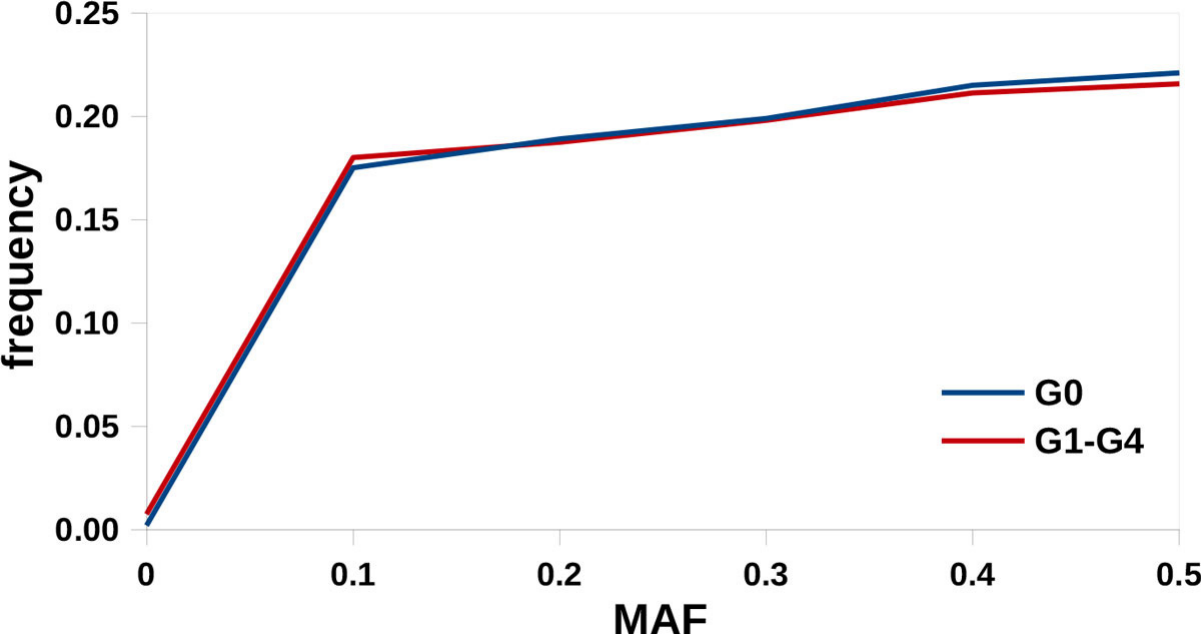
Minor allele frequency distribution in the simulated population

**Figure 2:**
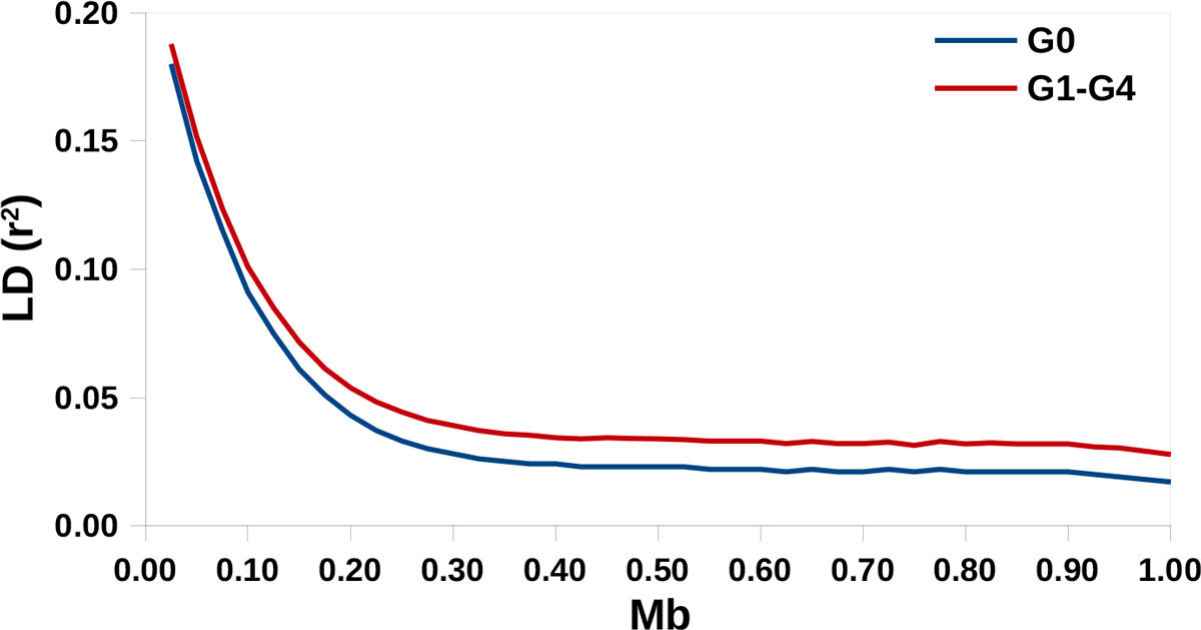
Linkage disequilibrium (r2) decay, as a function of the distance between markers, realized in the simulated population

The minor allele frequency (MAF) of simulated SNPs (Figure 1) and the realized LD was typical of ovine populations (Figure 2) [12,13]. The average r^2 ^obtained at distances larger than 100 kb was lower than that observed by other authors on dairy sheep populations because no relationships were simulated among G0 individuals. This condition is almost impossible in real populations, particularly if they are under selection. The average distance between adjacent QTLs was 8.62 Mb, ranging from 0.10 to 46.60. The r^2 ^between adjacent QTLs averaged 0.013, with a maximum of 0.13. Table 2 reports a summary of the QTLs classified according to their pleiotropic nature. The value of ω was on average 0.80 and ranged from -1 and 4. All the simulated QTLs had non-zero effects on T1, 46 had non-zero effects on T2, and 19 had non-zero effect on T3. Finally, heritabilities (Table 3) and the phenotypic and genetic correlations (Table 4) realized in the simulated population (G1-G3) were comparable with those observed in true dairy sheep populations [14].

**Table 2:** Summary of the simulated QTL classified by their pleiotropic degree

		Proportion of variance^1^			Contribution to the genetic correlation^2^		
			
*ω*	n. QTLs	T1	T2	T3	r(T1,T2)	r(T1,T3)	r(T2,T3)
-1	4	0.02	0.02	0.23	-0.02	-0.06	0.07
0	4	0.23	0.00	0.55	0.00	-0.35	0.00
>0<1	7	0.17	0.08	0.09	0.12	-0.12	-0.08
1	31	0.48	0.61	0.00	0.54	0.00	0.00
>1	4	0.10	0.28	0.13	0.16	0.09	0.18
Total	**50**	**1.00**	**1.00**	**1.00**	**0.80**	**-0.45**	**0.17**

^1 ^2pqα^2^_x_/σ_x_^2^, ^2 ^2pqα_x_α_y_/σ_x_σ_y_

**Table 3:** Genetic parameters realized in generations G1-G3

Parameter	T1	T1	T3
*σ_p_*	177	9.5	0.024
*σ_g_*	104	5.6	0.018
h^2^	0.35	0.34	0.53

*σ_p _*= Phenotypic variance, *σ_a _*= Additive genetic variance, h^2 ^= heritabillity

**Table 4:** Phenotypic (above diagonal) and genetic (below diagonal) correlations realized by simulation in G1-G3

	T1	T2	T3
T1	-	0.81	-0.44
T2	0.80	-	0.15
T3	-0.46	0.16	-

### QTL detectability

**Figure 3:**
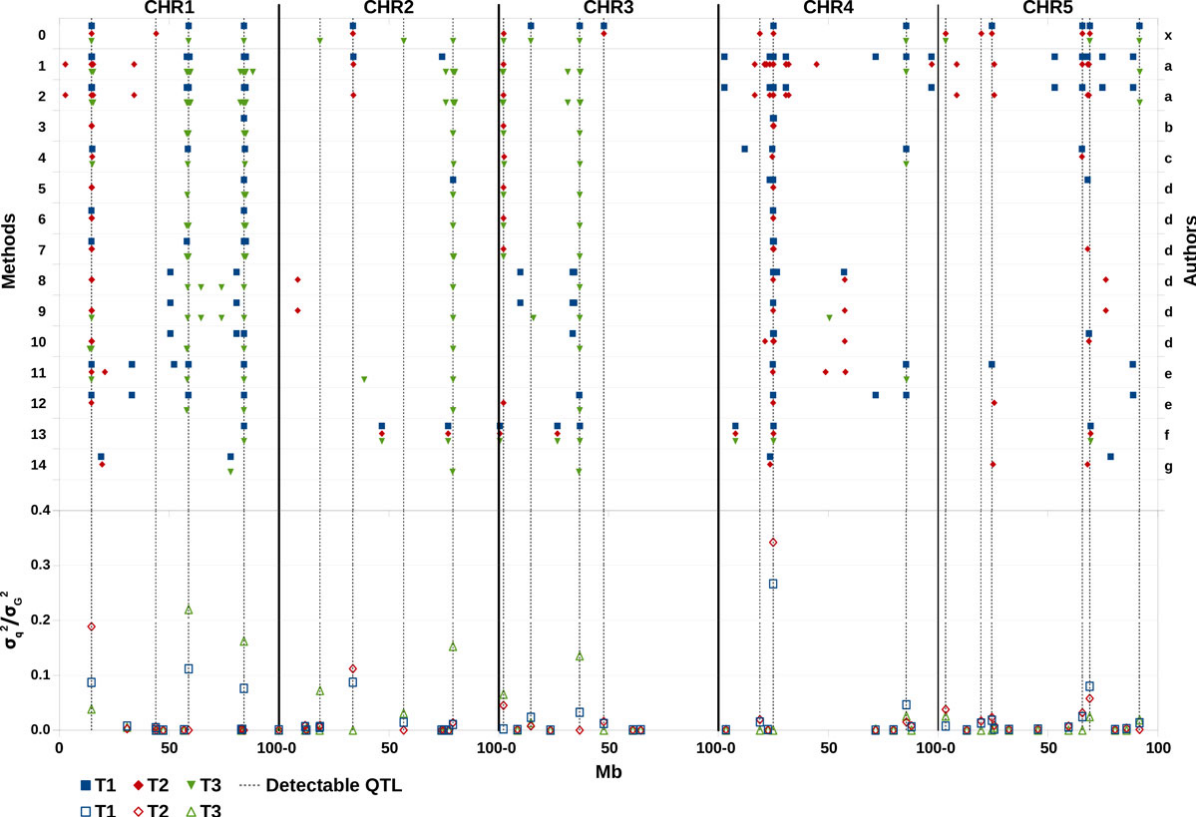
**QTL mapping results for methods tested in single-trait analysis and proportion of the whole genetic variance (σ^2^_G_) explained by each simulated QTL (calculated as 2pqα^2^/σ^2^_G_)**. Methods: 0- Detectable QTL; 1- RR_YD; 2- RR_YDadj; 3- GRAMMAR; 4- RHM20; 5- GRM_GC_MT; 6- GRM_GC_ST; 7- GRM_GC_YD; 8- RF_MT; 9- RF_ST; 10- RF_YD; 11- LDLA; 12 -DMU; 13- GEN-SEL; 14 LA. Authors: x- Organizers; a- Karacaroen; b- Grosse-Brinkhaus et al.; c- Riggio et al.; d-Minozzi et al.; e- Garzia-Gamez et al.; f- Moioli et al.; g- Demeure et al.

The detectability of the QTLs was, as expected, proportional to the genetic variance explained by each of them (Figure 3). Twenty-one of the 50 simulated QTLs were potentially detectable across traits. Within trait, 13, 12, and 13 QTLs explaining 0.88, 0.89 and 0.98 of the total genetic variance, were potentially detectable on T1, T2, and T3 respectively (Table 5). These QTLs generated a genetic correlation equal to 0.66, -0.39 and 0.13 between T1&T2, T1&T3 and T2&T3 respectively. Two QTLs were detectable on all the three traits; 5 on T1&T2; 6 on T1&T3; 2 on T2&T3; 3 on T2 only; and 3 on T3 only.

**Table 5:** Comparison of QTL mapping results

Method	False positive			True QTL			Proportion of genetic variance explained^1^		
	**T1**	**T2**	**T3**	**T1**	**T2**	**T3**	**T1**	**T2**	**T3**
			
RR_YD	9	15	5	8	6	8	0.78	0.78	0.82
RR_YDadj	7	8	4	5	5	7	0.57	0.75	0.79
GRAMMAR	0	0	0	2	3	5	0.34	0.58	0.74
RHM20	1	0	0	6	4	7	0.61	0.61	0.80
GRM_CG_MT	2	0	0	3	3	5	0.42	0.58	0.74
GRM_CG_ST	0	0	0	3	3	5	0.43	0.58	0.74
GRM_CG_YD	0	1	0	4	4	5	0.54	0.63	0.74
RF_MT	7	3	2	1	2	4	0.27	0.53	0.67
RF_ST	5	3	4	1	2	5	0.27	0.53	0.71
RF_YD	3	2	0	3	3	5	0.42	0.59	0.71
LDLA	3	3	1	6	2	5	0.61	0.53	0.60
DMU	3	1	0	6	3	4	0.62	0.58	0.67
SEL-GEN	5	5	6	4	2	3	0.45	0.40	0.32
LA	4	3	1	0	1	2	0.00	0.02	0.29

detectable QTL				13	12	13	0.88	0.89	0.98

^1 ^2pqα^2^_x_/σ_x_^2^

### Single-trait QTL detection

Table 5 shows a global view of the performance of the proposed methods for single-trait QTL detection. A detailed description of the proposed informative locations against the true QTL positions is given in Figure 3, where the detectable QTLs and proportion of genetic variance for each simulated QTL are reported. On the whole, RR [3] showed the highest detection power, particularly when applied to YD. Its application to phenotypes adjusted for LD structure resulted in some missed QTLs, in particular for T1. However, RR also produced a large number of false positives, especially for T1 and T2. The poorest results were for the LA approach [8]. In this case, however, most of the positions considered as false positives were in the same general region as the QTL, but were not close enough to the QTL to be counted as detections. The remaining proposed methods behaved differently depending on the trait analyzed. Results for T1 showed the largest variability. Indeed, for this trait the number of correct detections ranged from 1 to 6. The best results in terms of true detections were obtained by three rather different methods (DMU, LDLA and RHM20), all of which included a polygenic effect in the model. Among these, the best result in terms of type I error was obtained by RHM20 [4], with just 1 false detection. The lowest variability was observed among results for T2 and T3. RHM20 [4] and GRM_GC [5] performed on YD produced the best results for both of these traits.

The rate of successful detections, in general, agreed with the proportion of genetic variance explained by the true QTLs. Almost all of the participants were able to detect QTLs explaining more than 11% of the total genetic variance.

### Pleiotropic QTL detection

**Figure 4:**
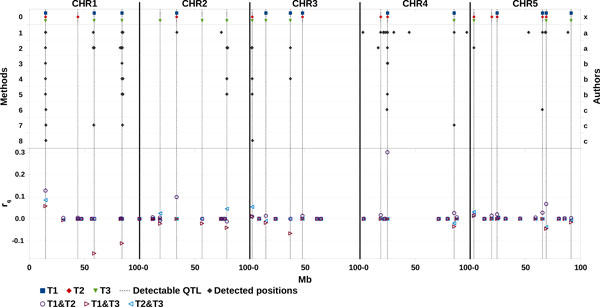
**QTL mapping results for methods tested in pleiotropic analysis and contribution of each simulated QTL to the genetic correlation (rG) between traits [calculated as 2pqα_i_α_j_/(σ_Gi_σ_Gj_)]**. Methods: 0- Detectable QTL; 1- RR_PC1; 2- RR_PC2; 3- GRAMMAR_PC1; 4- GRAMMAR_PC2; 5- Multivariate-Bayesian; 6- local EBV corr. T1&T2; 7- local EBV corr. T1&T3; 8- local EBV corr. T2&T3. Authors: x- Organizers; a- Karacaroen; b- Grosse - Brinkhaus et al.; c- Riggio et al.

Figure 4 reports the position of the identified pleiotropic QTLs and their individual contributions to the correlations between traits. Two authors [3,7] applied the same methods proposed for single-traits to the principal components extracted from the three original phenotypes. In both cases, the authors kept the first two PCs, which accounted for most of the variance. Karacaroen [3] performed RR on PCs extracted either from raw YDs or YDs adjusted for LD structure and obtained similar results in both cases; thus, only the results from YDs are reported here. RR [3] was able to detect 9 true QTLs when performed on the first PC (RR_PC1) and 8 true QTLs when performed on the second PC (RR_PC2). Four QTLs were detected on both PCs. Most of the detected positions had already been identified in the single-trait analysis, and several of the proposed positions were false signals. Two new positions were also identified, one by RR_PC1 and the other by RR_PC2. In general, the QTLs detected by RR_PC1 greatly contributed to the positive genetic (0.67) correlation between T1 and T2, while QTLs detected by RR_PC2 mainly affected the positive genetic correlation between T2 and T3. Three and five true QTLs were identified when GRAMMAR [7] was used on PC1 and PC2 respectively. Two QTLs located on CHR1 were identified in both of the principal components. No new significant SNP was added to those detected in the single-trait analysis. The QTLs detected on PC1 mainly explained the positive genetic correlation between T1 and T2, while those on PC2 were mainly related to the negative correlation between T1 and T3, and to the positive correlation between T2 and T3. The Bayesian multivariate approach [7] led to identify 5 true QTLs which generate a cumulative genetic correlation equal to 0.43, -0.13, and 0.18 for the T1&T2, T1&T3, and T2&T3 trait-pairs, respectively. The approach based on the correlation between regional EBVs estimated by RHM20 [4] identified 3 QTLs generating a positive T1&T2 correlation, 2 QTLs producing a positive T2&T3 correlation, and 4 QTLs contributing to the overall correlation between T1 and T3, one positively and 3 negatively. All of these regions agreed in sign with true covariance simulated at QTL level.

### Genomic predictions

The results of the comparison among GS prediction methods are presented in Table 6. Predictions for T1 were, in general, less accurate than those for T2 and T3. This result was expected since a larger amount of genetic variance was explained by fewer of QTLs in T2 and T3 compared to T1. The accuracy of prediction measured by r_DGV _ranged from 0.74 to 0.80, from 0.77 to 0.85 and from 0.76 to 0.84 for T1, T2, and T3, respectively. GBLUP provided lower accuracy than other methods. BayesA/B/C and Horseshoe method proposed by Pong-Wong [10] showed very similar accuracies for all traits, while the BayesLASSO produced slightly less accurate predictions. The best results in terms of accuracy were obtained by LASSO-based GRRM methods (GLASSO and sgLASSO). Among the other GRRM approaches, GSCAD gave accuracies very close to LASSO-based methods, while GBRIDGE and GMCP produced the poorest results, especially for T1 and T2. It is important to note that GRRM took advantage of marker grouping, which led to an increase in accuracy of about 0.10 [11], although the ideal group size was quite variable within trait and method.

**Table 6:** Comparison of predicted direct genomic values (DGV) with true breeding values (TBV)

			T1		T2		T3
**AU^1^**	**Methods**	**r_DGV_**	**b_DGV_**	**r_DGV_**	**b_DGV_**	**r_DGV_**	**b_DGV_**

PW	Horseshoe	0.79	1.06*	0.83	1.02	0.82	1.02
	BayesA	0.79	1.06*	0.83	1.03	0.83	1.03
	BayesB	0.79	1.06*	0.83	1.03	0.83	1.03
	BayesC	0.79	1.07*	0.82	1.02	0.82	1.01
	BayesLasso	0.77	1.11*	0.81	1.10*	0.79	1.03
	GBLUP	0.74	1.16*	0.77	1.16*	0.76	1.08*
OG^2§^	GBRIDGE	0.78	1.06	0.78	0.86	0.83	1.01
	GMCP	0.76	1.04	0.81	1.01	0.82	1.08
	GLASSO	0.79	1.25	0.85	1.22	0.84	1.20
	GSCAD	0.78	1.05	0.84	1.02	0.82	1.01
	sgLASSO	0.80	1.34	0.85	1.26	0.82	1.07

^1 ^Author: PW = Pong-Wong [10]; OG = Ogutu [11]

^2 ^Only the best results among group sizes were provided.

* significantly (p < 0.05) differs from 1

^§ ^significance could not be assessed

Unbiased estimators have regression coefficient (b_DGV_) of TBV on DGV of 1. In the present report, biases ranged from 0.86 to 1.34. All of the proposed methods overestimated TBV for all the traits except GBRIDGE, which underestimated the TBV of T2. b_DGV _significantly (p < 0.05) differ from 1 for all the methods implemented by [10] on T1, for BayesLasso and GBLUP on T2, and for GBLUP on T3 (Table 6). For the approaches proposed by [11], b_DGV _were more variable across traits and methods. However the significances of these values could not be assessed since predictions were not provided to the authors. The highest biases were observed in LASSO-based GRRM methods [11].

## Conclusion

The simulated data-set was provided to the participants in the XVI QTL-MAS workshop in order to compare QTL mapping and genomic selection approaches. The marker structure was similar to the SNP maps available in most livestock species with one SNP every 0.05 cM. The simulation procedure proposed here was very different from those commonly used in simulation studies. This novel approach was developed in order to more accurately representing LD of real livestock populations. In fact, obtained results were comparable with real data from sheep both in terms of MAF distribution and LD decay [12,13]. The genetic architecture for the quantitative traits was based on 50 segregating QTLs, of which only 12 to 13 had detectable effects, and the genetic correlations were based on QTL-specific covariances. As a result, generated genetic and phenotypic parameters realistically mimicked values estimated from field data.

Substantial variability was observed across methods either in terms of QTL detection power and Type I error. On the whole, the best results in terms of detection power were obtained by ridge regression, although it had a large number of false positives. This method was the only one that included a multilocus approach. Among the methods that performed a local test (one-by-one SNP testing or region testing) the best was RHM, in terms of detection power and Type I error, followed by DMU and GRAMMAR-GC. The latter method was used by two authors [5,7] on YDs but the obtained results differed either in the number of true and false detections. This is probably due to the different approaches that the authors used to assess test significance. Indeed, [5] used the nominal p-value, while [7] proposed a strategy based on permutations, which resulted in much more conservative results. When the same method was applied to different phenotypes for the same trait, YDs gave the best results. As expected, the poorest results were for the LA approach [8] due to the within-family linkage between QTLs located on the same chromosome, which generates interference among QTL effects.

In the pleiotropic analysis, some methods (RR) were able to identify some new positions missed in the single-trait analysis. However, the results did not permit clear determination of the nature of the pleiotropy underlying each QTL. A similar observation was also made for the multivariate Bayesian strategy. In fact, the strategy based on the analysis of correlations locally generated by each QTL [4] led to the correct interpretation of pleiotropic effects for each detected QTL, but did not identify any new QTL compared to single-trait analysis.

The accuracy of genomic predictions reported by the participants were quite similar across different methods. In particular, Bayesian methods and grouped regularized regression methods performed similarly. GBLUP did produce much lower accuracies, as expected from the literature. From a global evaluation perspective, methods that produce lower bias are preferred (BayesA/B/C/Horseshoes).

## Competing interests

The authors declare that they have no competing interests.

## Authors' contributions

MGU, AC and SC conceived the study and participated in its design; MGU programmed the simulations, performed comparative analysis and wrote the first draft of the manuscript related to QTL detection methods and results; GG performed comparative analysis and wrote the first draft of the manuscript related to genomic selection methods and results; NPPM, AC and SC critically revised the manuscript and contributed to discussion of the results. All authors read and approved the final manuscript.

## List of abbreviations used

GWA: genome wide association study; GS: genomic selection; SNP: single nucleotide polymorphism; QTL: quantitative trait locus; MAF: minor allele frequency; LD: linkage disequilibrium; TBV: true breeding value; YD: yield deviation; RR: ridge regression; RHM: regional heritability mapping; GRAMMAR-GC: genome wide association mixed model and regression - genomic control; SG: selective genotyping; IBD : identity-by-descent; PC: principal component; DGV: direct genomic value; RRM: regularizing regression method; GBLUP: genomic best linear unbiased prediction; GBRIDGE: group bridge regression; GMCP: group min-max concavity penalty; GLASSO: group least angle shrinkage and selection operator; GSCAD: group smoothly clipped absolute deviation; sgLASSO: sparse group LASSO.

## Declarations

The Work was equally funded by the Italian Ministry of Agriculture (grant INNOVAGEN) and AGRIS-Sardegna, Department for Livestock Production Research.

This article has been published as part of *BMC Proceedings *Volume 8 Supplement 5, 2014: Proceedings of the 16th European Workshop on QTL Mapping and Marker Assisted Selection (QTL-MAS). The full contents of the supplement are available online at http://www.biomedcentral.com/bmcproc/supplements/8/S5
